# Supplementary material for: Evaluation of sted super-resolution image quality by image correlation spectroscopy (QuICS)
Source: Sci Rep. 2021 Oct 21;11:20782. doi: 10.1038/s41598-021-00301-x (PMC8531054; doi:10.1038/s41598-021-00301-x)
Supplement: Supplementary file 1 — Supplementary Information. [file 41598_2021_301_MOESM1_ESM.pdf]

Supplementary Information of

Evaluation of sted super-resolution image quality by image correlation spectroscopy (QuICS)

Elena Cerutti[1,2], Morgana D'Amico[1], Isotta Cainero[2], Gaetano Ivan Dellino[3,4], Mario Faretta[3], Giuseppe Vicidomini[5], Pier Giuseppe Pelicci[3,4], Paolo Bianchini[2], Alberto Diaspro[2,6], Luca Lanzanò[1,2,*]

[1]Department of Physics and Astronomy "Ettore Majorana", University of Catania, Via S. Sofia 64, 95123 Catania, Italy

[2]Nanoscopy and NIC@IIT, CHT Erzelli, Istituto Italiano di Tecnologia, Via Enrico Melen 83, Building B, 16152 Genoa, Italy

[3]Department of Experimental Oncology, IEO, European Institute of Oncology IRCCS, 20100 Milan, Italy

[4]Department of Oncology and Hemato-Oncology, University of Milan, 20100 Milan, Italy

[5]Molecular Microscopy and Spectroscopy, CHT Erzelli, Istituto Italiano di Tecnologia, Via Enrico Melen 83, Building B, 16152 Genoa, Italy

[6]DIFILAB, Department of Physics, University of Genoa, via Dodecaneso 33, 16143 Genoa, Italy

*Corresponding author: luca.lanzano@unict.it

a

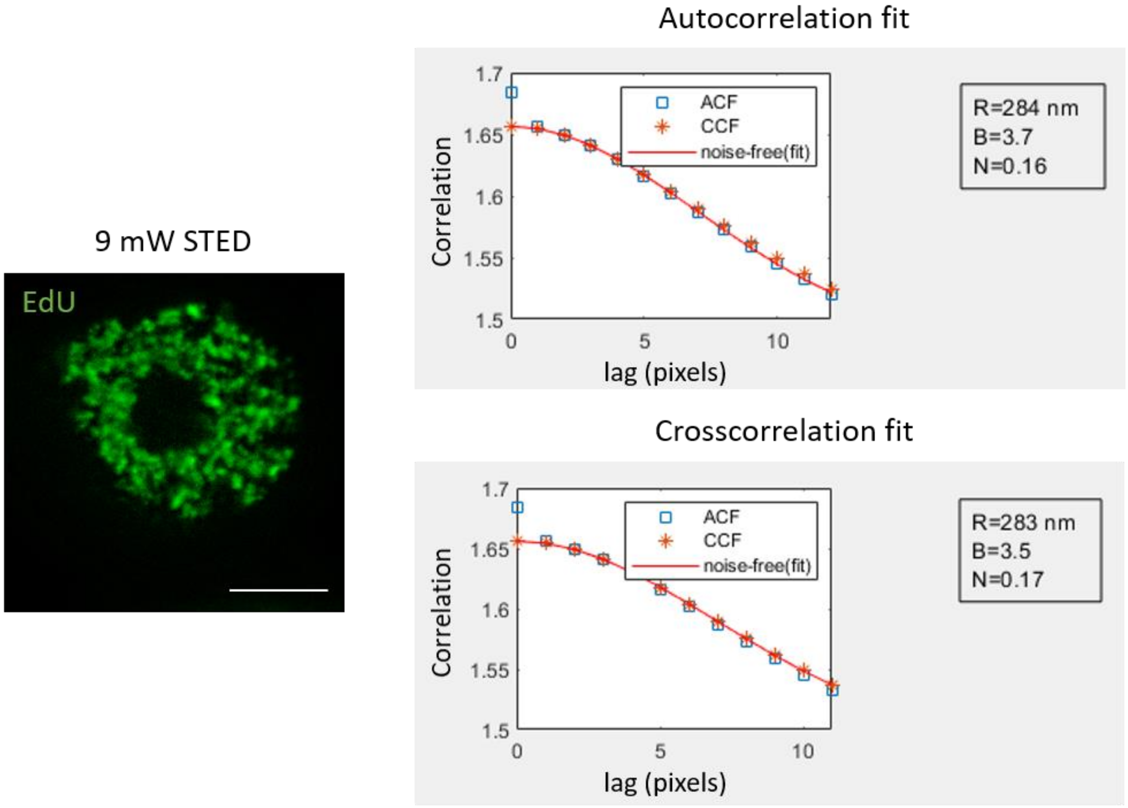
9 mW STED
EdU
Autocorrelation fit
ACF
CCF
noise-free(fit)
Correlation
lag (pixels)
1.7
1.65
1.6
1.55
1.5
0
5
10
R=284 nm
B=3.7
N=0.16
Crosscorrelation fit
ACF
CCF
noise-free(fit)
Correlation
lag (pixels)
1.7
1.65
1.6
1.55
1.5
0
5
10
R=283 nm
B=3.5
N=0.17

b

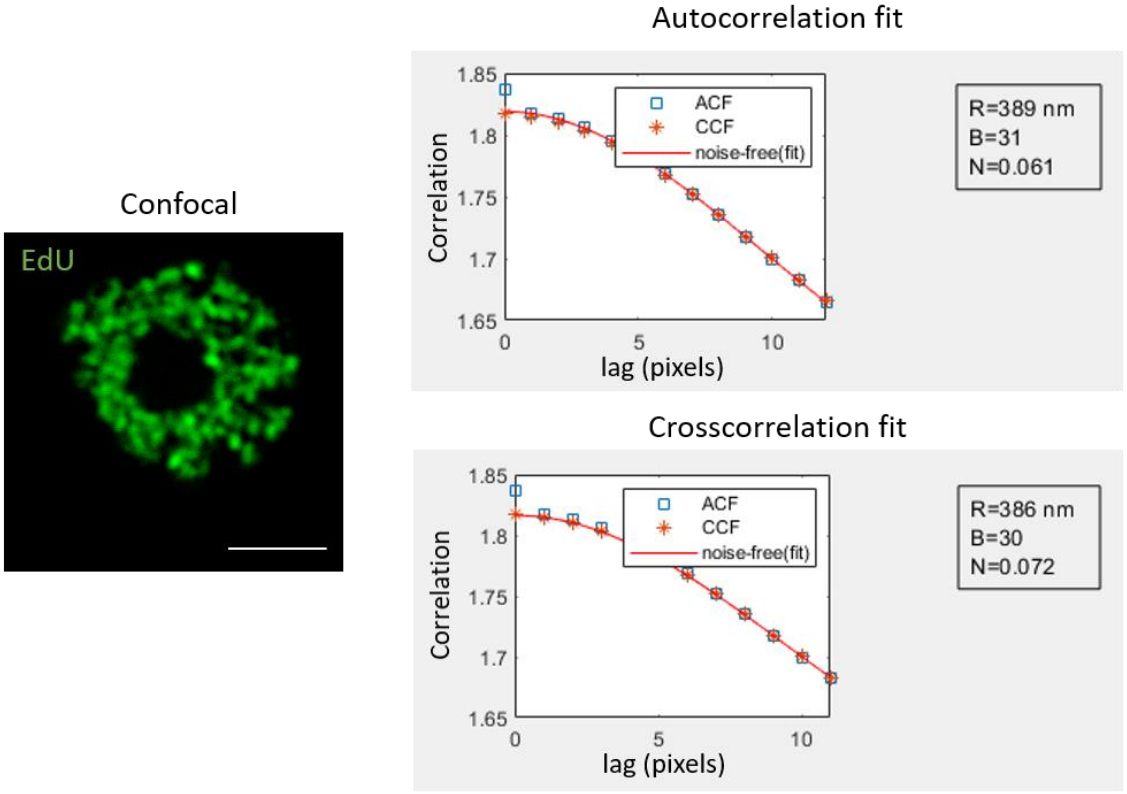
Confocal
EdU
Autocorrelation fit
ACF
CCF
noise-free(fit)
Correlation
lag (pixels)
1.85
1.8
1.75
1.7
1.65
0
5
10
R=389 nm
B=31
N=0.061
Crosscorrelation fit
ACF
CCF
noise-free(fit)
Correlation
lag (pixels)
1.85
1.8
1.75
1.7
1.65
0
5
10
R=386 nm
B=30
N=0.072

Supplementary Figure 1. Noise-free correlation function extraction

Shown is an example of data obtained with the QuICS algorithm from the same cell. **a** (left) STED image of U937-PR9 cell upon staining of DNA replication foci (STED depletion power 9 mW). (right top row) Fit of the autocorrelation function, excluding the zero lag point, and the relative extracted parameters: R, B, N. (right bottom row) Fit of the crosscorrelation function between two statistically independent images obtained through chessboard downsampling, and the relative extracted parameters. **b** Confocal image of the same U937-PR9 cell and QuICS analysis of the auto and crosscorrelation fits. Scale bars represent 3 µm.
